# Late-onset erythromelalgia in a previously healthy young woman: a case report and review of the literature

**DOI:** 10.1186/1752-1947-3-106

**Published:** 2009-11-04

**Authors:** Shobhana Gaur, Thomas Koroscil

**Affiliations:** 1 Department of Medicine, Emory University School of Medicine, Atlanta, GA 30322, USA; 2 Department of Medicine, Boonshoft School of Medicine, Dayton, OH 45408, USA

## Abstract

**Introduction:**

Erythromelalgia is a rare disorder characterized by episodic erythema and burning pain, which commonly involves the extremities. We present a case of late onset erythromelalgia in a previously healthy young woman and briefly review the literature. Our patient's case also has additional uncommon features not reported previously.

**Case presentation:**

A 33-year-old previously healthy Caucasian woman presented with complaints of episodic burning pain and flushing occurring in a central distribution involving her face, ears, upper chest and, occasionally, her upper extremities. Her symptoms were triggered by lying down or warm temperature exposure and were relieved by cooling measures. Extensive diagnostic work-up looking for secondary causes for the symptoms was negative and the diagnosis of erythromelalgia was made based on details provided in her clinical history supported by raised temperature in the affected area measured by thermography during a symptomatic episode. The patient did not respond to pharmacological therapy or surgical sympathectomy. She was advised on lifestyle modification to avoid activities which triggered her symptoms. She was hypothermic with a core temperature between 92 and 95°F. She also had premature ovarian failure, which had not previously been reported.

**Conclusion:**

Erythromelalgia is a rare disorder of unknown cause. There is no confirmatory diagnostic test; diagnosis is based on details provided in the patient's medical history and physical examination during the episodes. For those affected, this disorder leads to significant long-term morbidity and unfortunately, to date, no definitive therapy is available except for lifestyle modification.

## Introduction

Erythromelalgia (EM), also called erythermalgia by some authorities, is often characterized by episodic erythema, warmth and burning pain in the extremities. EM can be primary or secondary. Primary EM can be further divided into familial or sporadic of early (juvenile) or late (adult) onset. Secondary EM can be due to multiple causes including but not limited to myeloproliferative or autoimmune disorders and neuropathic conditions. Symptoms are triggered by physical exertion or a warm environment and can be relieved by cooling or elevation of involved limbs. Episodes may last from minutes to hours or even days. Early recognition of EM is important but difficult due to the rare nature of the disorder.

## Case presentation

A 33-year-old Caucasian woman presented with complaints of episodic burning pain and flushing of her face, ears, upper chest and, occasionally, her upper extremities. She presented with photographs displaying bright erythema from her lower neck extending upwards to her face and head. Her symptoms were precipitated and worsened with lying down or warm temperature exposure, and were abated by cooling measures. She maintained her home air temperature at 60°F. She denied similar symptoms amongst family members, and there was no family history of EM. She was in good health until 2001 when she began to experience these painful burning and flushing episodes.

Over time, the episodes increased in frequency and severity until presentation when she was experiencing multiple daily episodes, each lasting minutes to hours. Her symptoms were disabling and she was forced to leave her job. Physical examinations during multiple visits revealed normal vital signs except for a reduced core body temperature of 94° to 95°F. She had dry skin with a diffuse blanching erythema mainly over her face, and loss of her fingernails. She underwent an extensive evaluation which included a normal comprehensive metabolic panel and complete blood count (CBC) with differential. She did have a low free thyroxine level with a mildly elevated thyroid stimulating hormone level and was started on levothyroxine for primary hypothyroidism, but this did not change her symptoms. Approximately 1 year after the onset of symptoms, she developed oligomenorrhea and this later progressed to amenorrhea. She developed premature ovarian failure based on low estradiol levels and elevated luteinizing hormone (LH) and follicle-stimulating hormone (FSH). Ovarian ultrasound and pituitary magnetic resonance imaging (MRI) were normal. The autoimmune panel was normal including negative antiperoxidase, anti-adrenal, anti-ovarian, gastric parietal, antinuclear, anti-Smith, and anti-DNA antibodies.

Further testing revealed a mildly elevated serum tryptase level. A skin biopsy was performed, showing nonspecific changes. A normal bone marrow biopsy excluded systemic mastocytosis or myeloproliferative disorders. She was started on antihistamines/mast cell stabilizer, but this did not relieve her symptoms. A trial course of high-dose steroids was ineffective. She was also treated with intense pulsed light therapy without any success. Finally, the diagnosis of erythromelalgia was made based on her clinical history supported by raised temperature in the affected area during an episode, measured by thermography. Genetic testing was available, but it is not a confirmatory laboratory test. The patient was offered genetic testing, but she refused.

Our patient tried several medications including aspirin, Plavix (clopidogrel bisulfate), selective serotonin reuptake inhibitors (SSRI), tricyclic antidepressants (TCA), calcium channel blockers (CCB) and gabapentin. None of these medicines relieved her symptoms and only clonidine was temporarily modestly effective. Misoprostol was not significantly effective. Surgical sympathectomy was done, but was similarly ineffective and intravenous gamma globulin was tried without success. Recently, the patient has also tried a course of mexiletine. She started with 100 mg twice a day (BID) and this was increased to 200 mg BID after 2 weeks. She showed no improvement and the dose was increased 4 weeks later to 200 mg three times a day (TID). She noted some nausea but no other side effects. After a month, the dose was further increased to 300 mg TID for 4 weeks. Given the lack of clinical improvement, mexiletine was tapered and discontinued. Keeping her at home and very cool provided palliative relief and reduced the severity and frequency of the episodes. She pre-cools her car with remote starting during the summer and then sprints to the car to reduce her exposure to warm air. To date, she continues to avoid any activity that might precipitate any flushing and burning events.

## Discussion

Accurate incidence and prevalence of EM are difficult to calculate due to the patient's failure to recognize the condition when the symptoms are mild. Physicians may also fail to make the diagnosis as this is a rare and relatively unknown disorder. The proposed incidence is 2.5 to 3.3 per million per year with a prevalence of 18 to 20 per million in the Norwegian population [1].

In 1878, Mitchell [2] suggested the name 'erythromelalgia' because of the characteristic findings of redness (erythros) and pain (algos) involving the extremities (melos). Considerable confusion exists regarding the diagnostic criteria and nomenclature of EM [3]. Unfortunately, there is no confirmatory diagnostic test - the diagnosis must be made by taking a careful history, and can be supported by physical examination during the episodes. With the advent of digital photography, photographs can be very helpful to document the events of erythema. Thermography can reveal increased skin temperature in the affected area, but this is not necessary to establish the diagnosis.

Once a diagnosis is established, potential secondary causes must be excluded. CBC with differential is recommended for screening and follow-up. Other causes associated with erythromelalgia include drugs such as verapamil, nicardipine, bromocryptine, pergolide, and mercury poisoning, and diseases such as systemic lupus erythematosus, Raynaud's disease, pernicious anemia, thrombotic thrombocytopenic purpura, infectious mononucleosis and diabetic neuropathy. Appropriate testing should be considered based on diagnostic suspicion of the above conditions. Our patient underwent evaluation for neoplastic, connective tissue and autoimmune disorders and all results were normal.

The exact pathological mechanism responsible for this disorder is unknown, but several theories have been proposed to explain its pathophysiology. According to the microvascular arteriovenous (AV) hypothesis, symptoms are caused by tissue hypoxia induced by impaired distribution of skin microvascular blood flow with increased thermoregulatory flow through AV shunts and an inadequate perfusion. Work done by Mork and colleagues [4] supports this hypothesis of increased thermoregulatory flow through AV shunts during attacks in primary EM.

A prospective study done by Davis *et al *[5] suggested that EM is associated with a neuropathy, primarily small-fiber, and a vasculopathy (intermittent increased blood flow, AV shunting). There may be increased local cellular metabolism. However, it is unclear which one is the initiating event or primary abnormality.

An abnormal expression of the sodium channel has been linked with chronic pain, in particular, the Nav1.7 sodium channel isoform. The Nav1.7 channel is selectively expressed within the dorsal root ganglion (DRG) and sympathetic ganglia [6,7]. Yang *et al *[8] reported mutations in the SCN9A gene, which encodes the Nav1.7 sodium channel, in patients with primary EM. Cummins *et al *[9] demonstrated that these mutations in the Nav1.7 channel produce a hyperpolarizing shift in activation and slow deactivation causing the sodium channels to remain open for extended periods of time. Overall, these changes confer hyperexcitability on peripheral sensory and sympathetic neurons, which contributes to symptom production in primary EM. In 2005, Dib-Hajj *et al *[10] demonstrated another F1449V mutation in the Nav1.7 channel, which also reduces the firing threshold and produces abnormal repetitive firing in sensory neurons in primary EM. Nearly a dozen mutations in Nav1.7 have been identified. These mutations, found to be a cause of familial EM, can produce a hyperpolarizing shift in the activation of the channel. Please refer to articles by Dib-Hajj *et al *[11] and Drenth *et al *[12] for reviews on this topic. Lastly, the cause of sporadic EM remains unknown, however some juvenile cases reported were thought to be the result of spontaneous 'founder' mutations.

A universally effective treatment for EM is unknown. The mainstay of therapy is support and avoidance of trigger factors. Local measures include cooling or elevating the extremity to effectively attenuate or relieve symptoms. Patients should also be counseled about the use of safe cooling options such as fans and air conditioning. One must be careful using ice or immersing an extremity into an icy water bath to relieve symptoms, since this can lead to skin necrosis and ulceration.

Several case reports have shown a response to aspirin and intravenous administration of sodium nitroprusside [13] and prostaglandins. In secondary EM associated with myeloproliferative disorders, chemotherapy or phlebotomy has been used resulting in improvement of symptoms. Surgical sympathectomy has had variable results. Treatment with medications such as gabapentin, SSRIs, TCAs, and CCBs has had some symptomatic benefits in a few cases. One case study [14] reported the efficacy of sodium channel blockers such as lidocaine and mexiletine, however, our patient's symptoms did not improve with a trial of mexiletine therapy. She did have temporary improvement with misoprostol and clonidine, however beneficial effects were short-lived. She tries to control her symptom flares by keeping cool and setting her home temperature to 60°F.

A retrospective study of 168 patients with EM conducted at the Mayo Clinic showed significant morbidity and mortality [15]. Complications such as digital necrosis, skin ulceration, extremity gangrene leading to amputation and nail growth disturbances have been reported in a few cases. Near fatal hypothermia was reported in a single case related to constant cooling required to control the symptoms. Most concerning is the functional impairment reported by EM victims, such as inability to walk long distances, operate a motor vehicle or the inability to continue in their current job. Our patient remains unable to work and unable to tolerate any change in environment that exposes her to warm air.

Our patient's case has additional unusual features. Her pattern of involvement is mainly the upper chest, neck, face and head area, as opposed to the usual lower extremity involvement. She became hypothermic with a core temperature often in the 92-95°F range. This hypothermia is likely due to her chronic exposure and acclimation to a cool environment at home. An MRI scan of her brain was normal without any hypothalamic lesion, she had no other neurologic symptoms, and she hadn't suffered any head trauma. There was no prior history of body temperature dysregulation or poikilothermia. She had mild primary hypothyroidism with negative antithyroid antibodies. Lastly, she also developed premature ovarian failure, which has not been previously reported. We suggest that her low body temperature may be the cause of the premature ovarian failure.

## Conclusion

Erythromelalgia is a rare disease entity that leads to significant functional impairment. Our patient had typical and atypical features of EM. She probably suffered from a non-inherited form of erythromelalgia. Early recognition of this disorder and patient counseling are very important in order to minimize complications. Further research would be helpful to elucidate the underlying pathophysiology and to identify effective therapies.

## Abbreviations

AV: arteriovenous; BID: twice a day; CBC: complete blood count; CCB: calcium channel blockers; DRG: dorsal root ganglion; EM: erythromelalgia; FSH: follicle-stimulating hormone; LH: luteinizing hormone; MRI: magnetic resonance imaging; SSRI: selective serotonin reuptake inhibitors; TCA: tricyclic antidepressants; TID: three times a day.

## Consent

Written informed consent was obtained from the patient for publication of this case report. A copy of the written consent is available for review by the Editor-in-Chief of this journal.

## Competing interests

The authors declare that they have no competing interests.

## Authors' contributions

SG gathered the data, searched and reviewed the literature and drafted the manuscript. TK managed the patient, contributed significantly in conception and design, and critically revised the manuscript. Both authors read and approved the final manuscript.
